# Impacts of Fluoride Neurotoxicity and Mitochondrial Dysfunction on Cognition and Mental Health: A Literature Review

**DOI:** 10.3390/ijerph182412884

**Published:** 2021-12-07

**Authors:** Emily A. Adkins, Kelly J. Brunst

**Affiliations:** Department of Environmental and Public Health Sciences, University of Cincinnati College of Medicine, 160 Panzeca Way, Cincinnati, OH 45226, USA; brunstkj@ucmail.uc.edu

**Keywords:** prenatal/perinatal fluoride exposure, longitudinal fluoride exposure, developmental neurotoxicity, childhood/adolescent environmental health, mitochondrial function

## Abstract

This review focuses on the synthesis of current experimental and observational data regarding the effect of fluoride exposure on childhood mental health and the role of mitochondrial function as a mechanism of action. We aggregated data on the relationships between fluoride neurotoxicity, mitochondrial function, and cognitive and mental health using PubMed. Current animal and human research suggest that prenatal and perinatal fluoride exposure might have neurotoxic effects. These studies observed physical changes (fur loss and delayed reflex development in animals), intelligence loss, increased hyperactivity, and irregular moods associated with fluoride exposure. Two gaps in the literature were identified: (1) there is limited research on the mental and emotional impacts of fluoride exposure compared to research on cognitive outcomes, and (2) human studies primarily focus on prenatal and perinatal exposure, with little research conducted at other time points (e.g., adolescence). Furthermore, there is no agreed-upon mechanism for the neurotoxic effects of fluoride; however, fluoride can induce mitochondrial damage, including decreasing circulating mitochondrial DNA content, dysregulating biogenesis, and circular structure loss. Additionally, many neurodevelopmental conditions have mitochondrial underpinnings. More work is needed to elucidate the impact and timing of fluoride exposure on mental health and the role of mitochondrial function as a biological mechanism

## 1. Introduction

Fluorine is the ninth chemical on the periodic table; it is an anionic molecule that belongs to the halogen family. Fluorine is the most reactive and electronegative chemical that is currently known [1]. When fluorine acts as an ion or creates an ionic bond with another compound, it becomes known as fluoride [2]. Compounds containing fluoride exist naturally in trace amounts in human saliva, urine, and multiple tissues. Residential and industrial soil, air, and water have all been found to contain fluoride [1].

Fluoride naturally occurs in water. In many areas, natural water fluoridation occurs at low enough levels that it does not provide benefits in the form of enamel protection [3]. However, there are approximately 200 million people globally living in areas such as India, Iran, Kenya, and Mexico with endemic fluorosis due to water sources with high fluoride levels [4]. 

Manual water fluoridation in the United States dates to 1945 and was intended to increase fluoride exposure to reduce cavities in children. As of 2016, over 200 million people in the United States have access to fluoridated drinking water [5]. Furthermore, in the same year, United States Surgeon General Vivek H. Murthy described community water fluoridation as the most effective way to ensure that all members of a population have access to supplemental fluoride to avoid cavities and tooth decay [6]. 

Water fluoridation occurs in the form of adding either fluorosilicic acid (H2SiF6), sodium fluorosilicate (Na2SiF6), or sodium fluoride (NaF) to the water supply. Fluorosilicic acid is the most used compound in water fluoridation. Frequently, small water systems will use sodium fluoride instead [7]. The American Dental Association (ADA) recommends adding fluoride to water at a ratio of 0.7 parts per million, or 0.7 milligrams per liter (mg/L) [8]. The maximum amount of fluoride that the Environmental Protection Agency (EPA) will allow in public water systems is 4.0 mg/L [9]. A dose of sodium fluoride that is between 40–80 mg/kg can produce lethal toxicity in humans [10]. Due to the wide range of fluoride dosing, humans may be exposed to fluoride without acute effects, and determining the potential neurotoxic effects of chronic exposure has begun to take the forefront of fluoride-based research.

Fluoride exposure is thought to have both short-term and long-term effects, especially when exposure occurs during critical points in development. Researchers are concerned that chronic low-level fluoride exposure could lead to lifelong deficits in intelligence as well as future mental health issues [11,12,13]. Moreover, concern for this exposure grows as the exact pathogenesis for fluoride exposure is not currently known. While factors such as genetics and iodine levels in the body may impact the potency of exposure impacts [11,13], more research is needed to determine exactly what leads to susceptibility. Several published articles have postulated that fluoride could be producing alterations in mitochondrial DNA; mitochondrial DNA has many implications in various mental disorders [14,15,16,17,18,19,20,21,22,23,24,25,26,27,28,29,30,31,32].

Thus, the purpose of this review is to gather existing literature that is related to childhood fluoride exposure, mitochondrial function, mental health, and cognitive outcomes, while also reviewing potential biomolecular mechanisms. Sources focusing on both animal and human outcomes are utilized in this research. While much of the published literature that exists focuses on the cognitive effects of fluoride on children, significantly less literature exists that discusses the relationship between fluoride and mental health issues such as depression and anxiety. Even fewer studies have teased out possible mechanistic implications linking fluoride and neurodevelopment. The goal of this review is to consolidate data in this field and to help researchers identify the gaps and plan for future studies. 

## 2. Methods

Publications on fluoride neurotoxicity were identified using the PubMed database, and screening occurred on the basis that the papers contained information related to prenatal and/or childhood fluoride exposure and neurotoxic effects. Results were filtered to only include those articles that were relevant to fluoride exposure, and duplicate articles were removed. All articles were written in or translated to English and were published between 1981 and 2020. Most of the resources that were used for this review were published within the previous decade. Research articles based on per- or poly-fluoroalkyl substances were excluded, and only research looking at fluorosilicic acid, sodium fluorosilicate, and sodium fluoride was utilized. Moreover, studies discussing dental health, fluorosis, or comprehensive human health risk analyses were not used in this review, nor were studies that primarily focused on fluoride as a co-exposure. While cognitive outcomes relating to fluoride exposure have been studied by several researchers globally, there are very few studies (*n* = 9) looking at fluoride exposure on mood and mental health (*n* = 5 (animals) and *n* = 4 (humans)); thus, these were included in our review. While studies suggest that fluoride can alter mitochondrial function and since mitochondrial function has been independently linked to adverse neurodevelopment, studies assessing the role of mitochondrial function in fluoride-related neurodevelopmental outcomes are sparse. Therefore, we present a body of literature supporting this hypothesis.

Meta-analyses and reviews such as those conducted by Phillipe Grandjean [12,13], Anna Choi [11], and Rivka Green [33] have dissected multiple studies looking at animal and human outcomes resulting from prolonged fluoride exposure. While several meta-analyses have been conducted regarding the impact of fluoride on cognition, no reviews address mental health outcomes or the potential mitochondrial implications that could represent a mechanism for fluoride neurotoxicity. We briefly summarize the conclusions from prior reviews by Grandjean, Choi, and Green [11,12,13,33] and expand the review by including one additional animal study [34] and four additional human studies [35,36,37,38]. These studies present a variety of findings, some of which are inconsistent with one another and with previously published literature. A 2019 study of Wistar rats affirmed that fluoride may pass from the mother to the fetus, resulting in memory impairment [34]. In humans, recent research has highlighted associations between fluoride exposure and a reduced intelligence quotient (IQ) [36,37] and increased somatization behaviors [38], particularly in males. However, conflicting research suggesting the protective effects of fluoride on cognition was published as recently as October of 2021 [35]. Regardless of their findings, nearly all the researchers who have worked on recent studies call for further investigation into the potential neurotoxic risks of fluoride to ensure population safety.

## 3. Results

### 3.1. Fluoride and Neurodevelopment

#### 3.1.1. Animal Studies: Cognitive Function

Very few works exist that have studied fluorosilicic acid or sodium fluorosilicate toxicity, but several articles have focused on the effects of sodium fluoride. Sodium fluoride exposure in animals has been linked to cognitive, behavioral, and memory disruption [34] (Table S1: Sources related to developmental fluoride neurotoxicity). Based on their current stage of central nervous system development, the overall consequences of fluoride exposure varied in rats/mice with more severe neurotoxic effects being observed among still-developing rats and mice compared to their adult counterparts [39,40,41]. For example, fluoride exposure during critical periods of development has a statistically significant impact on mouse cognition and behavior such as slower spatial learning, depressive tendencies, and anxious tendencies [40]. Short-term and long-term memory impairment has also been noted [34].

Researchers also found that prenatal fluoride exposure in rats was associated with hyperactivity that was comparable to that induced by amphetamines [39]. Despite the differences in hyperactivity levels, weight and plasma fluoride levels were comparable between test and control subjects [39]. Behavioral alterations were also present in mice and rats; this is thought to be due to modifications to the still-developing blood–brain barrier [39,40] as well as to effects caused by oxidative stress [42,43]. Altogether, animal studies seem to indicate that repeated doses of fluoride can result in atypical cognitive outcomes when compared to controls.

#### 3.1.2. Animal Studies: Sex Differences

The behavioral repercussions of sodium fluoride exposure in rats are also specific to sex and dose. Sodium fluoride exposure has been found to have adverse effects on learning ability and memory in rats, with more significant effects being observed among male rats [44]. In contrast, maternal sodium fluoride exposure at a low level (5–10 mg/L) was correlated with reduced anxiety in the young female rats and the adult rats compared to young male offspring. Low doses of fluoride also yielded increased hyperactivity in adult offspring born to exposed mothers [41]. Furthermore, many animal studies have linked fluoride to an increase in ADHD that is especially prevalent in males [39,40,44]. There are limited data regarding sex differences in animals that have been exposed to fluoride, and further research is needed to elucidate any potential relationships that may exist in this domain. 

#### 3.1.3. Animal Studies: Behavior

Fluoride exposure may also cause behavior-related effects in mice, with several studies suggesting an imbalance between nervous system excitation and inhibition depending on the dose and exposure time to fluoride [45]. Over time, fluoride-exposed mice may exhibit increased serotonin levels compared to non-exposed mice. Mice that have been exposed to fluoride have been shown to exhibit increased serotonin and brain fluoride levels at multiple time points following the exposure [46]. Serotonin (5-hydroxytryptamine [5-HT]) is a neurotransmitter that when present in the brain at inadequate levels, is heavily implicated in the development of depression and anxiety [47]. For example, 5-HT1A agonists, 5-HT1 antagonists, and 5-HT2 antagonists have been indicated for use in the treatment of many forms of anxiety disorders [48]. 

Fluoride exposure has also been linked to increased excitement in the hippocampus, impaired memory, anxiety-like behavior, and depression-like behavior in adult mice [49,50]. This may be due to the passage of fluoride through the blood–brain barrier, causing maladaptive changes to the hippocampus [45]. These animal studies suggest that fluoride may be impacting behavioral outcomes such as anxiety and depression through alterations in serotonin levels and/or via changes in specific regions of the brain in response to fluoride exposure (e.g., hippocampal excitement) [45].

#### 3.1.4. Human Studies: Cognitive Function and Previous Analyses

There is much debate on the topic of fluoride as a human neurotoxicant [9,10,11,51,52]. Developing brains are significantly more susceptible to neurotoxic damage from fluoride than mature brains are [13,37,53]. Children have a higher fluoride retention rate than adults; adults typically retain 50–60% of ingested fluoride, while infants and children retain approximately 80–90% [13]. This has led researchers to explore the impact that fluoride has on brain development. The first wave of manuscripts, as summarized by previously published reviews and meta-analyses, focused on cognitive outcomes, and the findings suggested that fluoride exposure can lead to a lower IQ in developing children [11,12,13].

There is a significant body of research assessing the cognitive outcomes resulting from fluoride exposure. Since 2012, many journals have published several notable literature reviews and meta-analyses regarding fluoride and developmental neurotoxicity. A meta-analysis conducted by Choi et al. in 2012 looked at twenty-seven studies published between 1989 and 2011. This analysis concluded that increased fluoride exposure was related to a decreased IQ in children. Additionally, it was determined that children may be at an increased risk of neurotoxic outcomes from fluoride when compared to their adult counterparts. Due to limitations such as missing information, potential misclassification bias, and methodological deficiencies, this review stated that more robust data were needed to further conclude the impact that fluoride has on neurodevelopment [11].

Phillipe Grandjean, a prominent researcher in developmental toxicity, has written two reviews since 2014 analyzing various cross-sectional and ecological studies relating to fluoride exposure [12,13]. The 2014 review by Grandjean builds upon a previously published analysis of industrial chemicals that could act as developmental neurotoxicants, adding fluoride to the list of compounds needing further investigation. This work reaffirmed Choi’s analysis, finding that fluoride may act as a neurotoxicant during developmental periods. An updated review by Grandjean that specifically focused on fluoride was published in 2019. Here, Grandjean looked at fourteen cross-sectional studies on the association between fluoride exposure and intellectual disability. His analysis of these studies indicated that safe doses of fluoride may be below the currently recommended levels for most water supplies [13]. 

Further, Green et al. reviewed 138 animal studies and 106 human studies for evidence of sex-specific, fluoride-based neurotoxicity in 2020 and found that males may be more affected by prenatal fluoride exposure. This relationship was not observed in postnatal fluoride exposure [33]. While not all studies included in this review supported the idea of increased risk of negative outcomes to males resulting from fluoride exposure, it is an area that still warrants further investigation.

#### 3.1.5. Human Studies: Mental Health and Neurobehavior

Increased fluoride levels in tap water have been associated with increased ADHD clinical diagnoses and symptoms such as hyperactivity and inattention. However, urinary fluoride levels are not found to predict ADHD diagnoses or symptoms [54]. These data indicate that prenatal fluoride exposure may be a critical period for exposure and that it may result in delayed behavioral effects [55]. 

Furthermore, despite the suggestive findings among animal studies, only one human study has investigated the impact of fluoride on mental health outcomes, such as anxiety and depression, in children or adults. Statistically significant findings have associated urinary fluoride content with somatization behaviors. However, this relationship was not observed in depression- or anxiety-like behaviors, which was unexpected due to their typical comorbidity with somatization [38].

#### 3.1.6. Human Studies: Sex Differences

Sex differences are also noted in some cognitive human studies of fluoride exposure, though this has not yet been widely researched. Males seem to be more susceptible to endocrine-disrupting chemicals, leading some researchers to believe that fluoride could have a more significant impact on male cognition and mental health than female cognition [12,13]. Additionally, critical windows of exposure to fluoride may vary based on sex; some data indicate that the prenatal window may be more critical for males, while the infancy window may be more critical for females [37]. Males seem to show a more significantly lowered IQ than females in studies looking at equivalent maternal urinary fluoride levels in both sexes [4,10,12,56]. This trend has also been observed based on maternal fluoride intake from food [57]. However, some studies exploring sex differences saw null effects. These include studies in the U.S. and Canada.

In the United States and Canada, exposure to artificially fluoridated water has been associated with a higher prevalence of ADHD in children [54,55,58]. The emerging research assessing sex, fluoride exposure, and mental health is conflicting, with some data suggesting opposing or null findings [35,54]. 

In contrast, research on internalizing symptomology in adolescents that have been exposed to fluoride found that males showed an increased likelihood of somatization symptoms [38]. Male children were at a nearly sevenfold greater risk of elevated overall internalizing symptomology compared to their female counterparts; a similar relationship was observed with somatization-like symptoms, but this relationship was not shown to be significant with depression- or anxiety-like symptoms [38].

### 3.2. Mitochondrial Dysfunction and Other Potential Pathogenesis of Fluoride

Several studies have linked fluoride exposure to mitochondrial dysfunction (Table S2: Sources related to fluoride toxicity and mitochondrial function) [14,15]. Mitochondria are energy-producing, membrane-bound organelles that produce most biochemical reactions within eukaryotic cells. Mitochondria serve a variety of purposes, including regulating metabolism and apoptosis [16]. They contain a form of DNA that is known as mitochondrial DNA (mtDNA); mtDNA is primarily inherited maternally [17]. Mitochondrial DNA is known to have high rates of mutations, many of which are linked to diseases such as cancer, diabetes, and several neurodegenerative disorders [16]. While many studies of both animal and human responses to fluoride exposure have found evidence of neurotoxicity, a mechanism of this damage is not universally agreed upon. Some studies attribute an association between neurotransmitter levels and fluoride consumption to claims of neurotoxicity, others emphasize changes in neuroanatomy, and others suggest mitochondrial dysfunction as a potential mechanism.

#### 3.2.1. Animal Studies: Fluoride and Mitochondrial Structure Changes

Chronic exposure to fluoride in rats can cause neuronal dysfunction and structural changes: this may alter rates of fission and fusion. Lowered levels of circulating mitochondrial fusion and fission-related particles are associated with intellectual loss in children who have been subjected to chronic fluoride exposure. Therefore, monitoring circulating mitochondria levels could provide insight into fluoride neurotoxicity and cognitive defects. Scientists must conduct more research to determine the effectiveness of this method [14]. Hippocampi that have been extracted from rats whose mothers were exposed to fluoride were found to have lower relative mtDNA levels compared to controls [19]. 

#### 3.2.2. Animal Studies: Fluoride: Mitochondrial Damage and Neuroinflammation

There are multiple pathways of interest for researchers studying fluoride as a potential neurotoxicant. Damage to the mitochondria resulting in mitochondrial dysfunction is one mechanism that is currently under investigation. In mice, sodium fluoride concentrations of about 5 mg/L have been shown to lead to oxidative stress and the inhibition of antioxidant enzymes [18]. This leads to higher concentrations of reactive oxygen species (ROS), causing mitochondrial damage, including lipid peroxidation, mitochondrial membrane depolarization, and cell apoptosis. ROS can also result in the degradation of mtDNA [18]. Furthermore, sodium fluoride is suspected to lead to autophagy deficiency, apoptosis augmentation, compromised neuronal survival, membrane loss, increased permeability, and reduced oxidative phosphorylation in the mitochondria of rats [14].

Separately, various causes of neuroinflammation are also of interest to researchers. Neuroinflammation may occur because of fluoride intake, resulting in potential neurological and neurodegenerative disorders based on the dose of fluoride received. Rats that have been chronically exposed to sodium fluoride exhibited higher expression of cyclooxygenase COX2, vascular endothelial growth factor (VEGF), and heat shock protein-70 (HSP-70), which act as markers of neuroinflammation. This relationship appeared to be dose dependent [20]. Additional rat data have indicated that cyclooxygenases, including COX1 and COX2, were reduced in the brains of rats that had been subject to chronic fluoride exposure. PGE2 concentration was also markedly increased, giving further weight to the hypothesis that neuroinflammation may result from fluoride exposure [59].

#### 3.2.3. Animal Studies: Fluoride, Neurotransmitters, and Signaling Pathways

Fluoride consumption may also impact neurotransmitter levels [20]. Serotonin levels have been shown to significantly increase in the brains of rats following fluoride exposure Notably, between 60–100 ppm of NaF, increases in serotonin have been observed at an above dose-dependent level. Glutamate and histamine levels have been shown to increase as well, while acetylcholine and dopamine levels have been shown to decrease. Irregularities in neuroanatomy such as swollen mitochondria, disrupted myelin sheaths, enlarged axons, and vacuolated Schwann cells have been exhibited by rats [20]. 

In Neuro-2A cells in mice, similar outcomes were observed. Cells that had been exposed to fluoride for 24 h were observed to have swelling, agglutination, vacuole formation, edema, and loss of synapses compared to controls. The release of the neurotransmitter glutamate was disrupted, and structural damage occurred within the cell, supporting the idea of fluoride-induced neurotoxicity [21]. 

Another pathway that is under investigation for fluoride-induced effects is the PGC-1α/NRF1/TFAM pathway. The PGC-1α/NRF1/TFAM signaling pathway is key in regulating mitochondrial biogenesis, and it may be significantly impacted by fluoride exposure [19,22]. Silent information regulator 1 or sirtuin 1 (SIRT1) is a protein that is important in the process of mitochondrial biogenesis, as it deacetylates PGC-1α (peroxisome proliferator-activated receptor γ coactivator-1α) [19,22]. In turn, the activation of PGC-1α begins the transcription of NRF1 (nuclear respiratory factor 1), which binds to a promotor site to regulate TFAM (mitochondrial transcription factor A) transcription [19,22,23]. TFAM is an essential component in mitochondrial replication, as it recognizes promoter sites, promotes transcription, and can unwind and conduct maintenance on DNA strands [22,24]. 

Fluoride is thought to potentially mediate the acetylation of the p53 tumor suppressor, which has been demonstrated by LS8 cells [25]. The p53 pathway is important in regulating apoptosis, and hyperactivation has been linked to many issues such as multiple sclerosis, arthritis, and neuropathies; the acetylation of p53 may contribute to its activation. Furthermore, mitochondria are key in helping this pathway to function properly, controlling the release of multiple important apoptogenic factors [26]. As SIRT1 is a histone deacetylase, it can lead to the deactivation of p53 to decrease apoptosis [25]. Fluoride exposure can lead to the generation of reactive oxygen species, resulting in mitochondrial damage. This may also contribute to p53 acetylation and promote apoptosis. SIRT1 may utilize PGC-1α to protect against fluoride-based mitochondrial damage [27]. 

**Figure 1:**
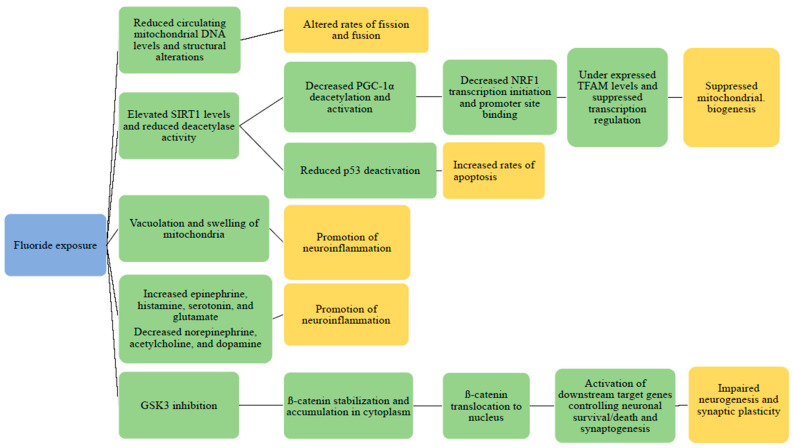
**Potential mechanisms of fluoride-based mitochondrial damage.** The proposed impact of fluoride exposure is detailed regarding the PGC-1α/NRF1/TFAM signaling pathway, p53 deactivation, alterations in neurotransmitter levels, and fission/fusion levels in mitochondria. Fluoride exposure is highlighted in blue; mechanisms of mitochondrial damage are highlighted in green, and outcomes are highlighted in yellow.

The glycogen synthase kinase 3ß (GSK-3ß)/ß-catenin signaling pathway may be compromised by fluoride exposure, resulting in potential neuronal death or apoptosis [59]. Sodium fluoride, in a dose-dependent manner, is thought to weaken neurogenesis in rats based on this pathway. When fluoride was introduced to rats at a high dose, it was found that GSK-3ß activity was induced, resulting in reduced downstream ß-catenin signaling. ß-catenin was also decreased in the nucleus and had reduced cytoplasmic expression. This could be interpreted as a sign of neurotoxicity [60]. The elucidation of this mechanism can be seen in Figure 1.

Mitochondrial function may also impact the nucleus accumbens (NAc) portion of the brain. The malfunctioning of this portion of the brain has been linked to depression [28]. The NAc also seems to be altered in animal models showing anxiety disorders [61]. Rats showing lower levels of adenosine triphosphate (ATP) in the NAc are more likely to show anxiety; lower levels of ATP availability may indicate mitochondrial dysfunction [30]. 

#### 3.2.4. Human Studies: Role of Mitochondrial function in Mental Health

##### Mitochondrial Volume

Human fetal brain samples in areas with fluorosis have a significantly lower volume and density of mitochondria compared to those that have not been exposed [14]. A fluoride study on Chinese children illustrated how low-to-moderate water fluoride and urinary fluoride levels show an inverse association with mtDNA levels (a marker of mitochondrial dysfunction) [32]. A 1 mg/L increase in the water fluoride concentration was correlated to a 0.10-unit decrease in relative mtDNA levels. Furthermore, a 1 mg/L increase in urinary fluoride concentration was correlated with a 0.12-unit decrease in relative mtDNA levels. Interestingly, the effects of fluoride exposure had a more severe impact on male children than on female children [32].

##### Mitochondrial Swelling, Autophagy, and Apoptosis

Mitochondrial swelling, autophagy, and apoptosis because of fluoride exposure have all been noted in multiple studies [21,32,58,62]. Human neuroblastoma SH-SY5Y cells that have been chronically treated with fluoride have shown altered morphology, including elongation of the mitochondria, swelling, and cristae disorders observed via transmission electron microscopy. These significant structural changes indicate that fluoride exposure could lead to neurotoxicity [15].

Altered fission and fusion rates in mitochondrial dysfunction are also a source of interest in those studying fluoride-based damage. Fluoride neurotoxicity could be caused by reduced autophagy and superfluous apoptosis linked to mitochondrial fission/fusion activity. When mitochondrial fission was inhibited in SH-SY5Y cells that had been damaged by fluoride exposure, researchers observed an increase in apoptosis and defective autophagy compared to fluoride-damaged cells with regular fission levels [14]. 

Moreover, several studies have shown that SH-SY5Y cells experience changes in apoptotic rates and mitochondrial function when exposed to fluoride [19,63]. Researchers looking at fluoride-based neurodevelopment damage determined that fluoride-treated SH-SY5Y cells showed links to a decrease in mitochondrial biogenesis. PGC-1α, NRF1, TFAM, and total mtRNA levels were reduced. This research supports the idea that the SIRT1-dependent PGC-1α/NRF1/TFAM signaling pathway regulates mitochondrial biogenesis and contributes to developmental neurotoxicity when hampered by chronic fluoride exposure [19].

The mitochondrial p53 apoptotic pathway has also been investigated in SH-SY5Y cells, with SIRT1 potentially serving as a protective agent against damage from fluoride. SH-SY5Y cells were exposed to fluoride for 24 h and infected with a SIRT1-encoding adenovirus, while the control cells were not exposed to the adenovirus. Deacetylation by SIRT1 was measured via an assay kit, and researchers determined that the apoptotic rates of cells were significantly increased at doses such as 40 and 60 mg/L NaF. These doses of fluoride also highlighted the nuclear translocation of p53. The inhibition of p53 transcriptional activity reduced the apoptotic rate in NaF-treated cells, implicating the p53 pathway in the apoptosis levels that were related to fluoride exposure. SIRT1 activity was also decreased in cells that had been exposed to NaF [62]. 

Mitochondria have a potential link to mental health disorders such as depression, anxiety, and ADHD [28,29,30,31,61]. Different studies imply several methods of pathogenesis, linking mitochondrial function to mental health. For example, while the exact mechanism causing depression is unknown, it is suspected that depression could be partially due to a lack of energy to carry out cellular processes and neuronal communication. Mitochondria also play a role in neurogenesis and developing neuroplasticity; stress can lead to decreased neurogenesis and can potentially contribute to depression symptoms [28]. Furthermore, mitochondrial energetics and its implication in ADHD development is a topic with emerging, though limited, data. Mitochondrial dysfunction leading to a decrease in ATP production may be related to a proposed bioenergetic crisis within ADHD [31]. 

#### 3.2.5. Human Studies: Areas with Limited Mitochondrial Research

Within the fields of fluoride neurotoxicity, mitochondrial function, and environmental factors on mental health, there are several notable gaps in the available literature. A significant amount of literature exists on the effects of fluoride compounds on cognition [4,11,12,13,20,58,64]. However, the impact of this chemical on mental health has been studied less widely. Various research studies have looked at depression, anxiety, and stress in rats and mice due to fluoride exposure [39,40,42,44,45,46]. However, there is currently only one human-based study [38]. This research found statistically significant associations between fluoride and internalizing composite and somatization scores in children on the Behavior Assessment System for Children-2 (BASC-2). However, this study was cross-sectional and called for further examinations of this topic. Most articles on fluoride toxicity studied sodium fluoride as the source despite fluorosilicic acid being the most utilized source of water fluoridation [7]. Toxicity research on fluorosilicic acid or sodium fluorosilicate exists in a limited capacity. Pharmacokinetic research, however, has found no significant differences between sodium fluoride, sodium fluorosilicate, and fluorosilic acid in terms of maximum fluoride concentrations, the time to reach those maximum concentrations, or the six-hour area under the time–plasma concentration curves [65]. There are currently no published studies assessing the neurotoxicity of these compounds.

Furthermore, while several studies have linked fluoride toxicity to mitochondrial dysfunction and while several studies have linked mental health outcomes to mitochondrial dysfunction, there is little knowledge on how fluoride-related mitochondrial function impacts cognition and neurobehavior. Criticisms of fluoride neurotoxicity studies primarily take issue with the weaknesses of study designs or confounding factors that they believe may alter the results [52,66]. Details on studies discussing the impact of fluoride on mitochondria can be found in Table S2: Sources related to fluoride toxicity and mitochondrial function.

### 3.3. Recent Toxicology Research

In 2016, the National Toxicology Program published a report entitled “Systematic Literature Review of the Effects of Fluoride on Learning and Memory in Animal Studies”. A “low-to-moderate level-of-evidence” was found associating learning and memory disorders with fluoride consumption in rodents. This report did not consider the findings to be directly comparable to potential human health outcomes. Specifically, this report highlighted the need for continued research in this field with emphasis on sex differences, varied developmental stages, and the resulting changes in neuropathology due to chronic fluoride exposure [67].

The NTP published a monograph in 2019 titled “Systematic Review of Fluoride Exposure and Neurodevelopmental and Cognitive Health Effects”, which has led to controversy within the scientific community and has undergone several rounds of revision as a result. This report intended to review both animal and human studies to evaluate the association and mechanism between fluoride exposure and both neurodevelopmental and cognitive outcomes. Their literature review included 149 human-based studies, 339 non-human mammalian studies, and 60 in vitro or mechanistic studies, some of which overlapped. Based on a review of neurodevelopmental and cognitive factors measured via methods such as IQ, general cognitive index (GCI), and mental development index (MDI), the National Toxicology Program (NTP) declared moderate confidence in the association between fluoride exposure and neurodevelopmental impacts in children. They reported a low confidence level in this association in adults [68]. 

A review of this monograph was published by The National Academies of Science, Engineering, and Medicine in 2020. This review cited inconsistent risk-of-bias criteria for study inclusion in the monograph as well as the insufficient evaluation of confounders and overly broad conclusions as some of the main reasons as to why The National Academies deemed the findings of the monograph inadequate. The National Academies recommended conducting a meta-analysis of the data from the monograph before reanalysis [69]. 

The NTP published a revision to this monograph in 2020 [68], which was met by further commentary from the ADA and the National Academies as recently as 2021. Criticisms of this update include unclear meta-analysis methods, the improper wording of conclusions, and concerning inconsistency and the unavailability of certain data. The National Academies recommends additional updates to improve clarity. The ADA has also requested that fluoride be reclassified in the National Toxicology Program from “presumed neurotoxin” due to a lack of research in this field [70]. 

## 4. Discussion

Studies testing the effects of chronic fluoride exposure on rats and mice have consistently found associations with decreased intelligence and increased anxiety and hyperactivity when compared to controls. Human studies have also indicated that chronic fluoride exposure could have long-term neurotoxic effects on children who have been exposed during development, including decreased intelligence or the increased prevalence of ADHD. More research is needed to determine if there is a link between chronic fluoride exposure and depression- and anxiety-like symptoms in humans. Presently, very few studies (all animal) have looked at mental health outcomes such as depression or anxiety. Fluoride is known to have some positive effects on the body, including the prevention of dental caries; however, the chemical is also known to collect in brain tissue over time [23,32,57]. Further assessment is needed to determine if the benefit of dental protection outweighs the potential neurotoxic effects.

Many publications call for more in-depth research on the neurotoxic effects of fluoride, with a particular emphasis on controlling for key confounding factors such as socioeconomic status, parental IQ and mental health, area of residence, and other chemical exposures [54,55,71]. 

Further research is also warranted to determine if fluoride damage to mitochondria could lead to cognitive or mental health effects. Mitochondria can be altered in structure and function when repeatedly exposed to higher doses of fluoride than those that occur naturally. Mitochondria have also been linked to the development of mental disorders such as depression and anxiety, with additional implications in the development of ADHD.

## 5. Conclusions

Fluoride exposure is ubiquitous; fluoride is naturally found in most water sources in low amounts [4]. The addition of supplemental fluoride in water systems could have developmental detriments to regular consumers or the offspring of those who have been exposed. A review of the current literature was necessary to aggregate information related to cognition/mental health, mitochondrial function, and toxicity issues to increase accessibility and to prompt further work. In animals, fluoride has been implicated in altered cognitive functions, behavioral changes, and mitochondrial damage. Sex differences have also been noted in some studies addressing fluoride exposure in animals. Likewise, researchers continue to investigate cognitive, mental health, and mitochondrial abnormalities as they relate to chronic fluoride exposure. Chronic exposure may be linked to decreased intelligence, memory deficits, learning difficulties, and ADHD. There is little work on the effect of fluoride on internalizing disorders such as depression and anxiety. Additionally, we know very little about the impact of fluoride exposure during childhood and adolescence; current works primarily identify developmental deficits, only referencing prenatal and perinatal exposure. 

Fluoride-induced mitochondrial damage may include structural changes, damage resulting in neuroinflammation, altered neurotransmitter levels, and disruptions to key signaling pathways. Furthermore, the pathogenesis of fluoride exposure on mitochondrial function must be further evaluated. More work is needed to gain a comprehensive picture as to the impact and timing of fluoride exposure on mental health outcomes and the role of mitochondrial function as a biological mechanism.

## Supplementary Materials

The following are available online at https://www.mdpi.com/article/10.3390/ijerph182412884/s1, Table S1, Sources related to developmental fluoride neurotoxicity. Table S2, Sources related to fluoride toxicity and mitochondrial function.
